# Local Relationship between Global-Flash Multifocal Electroretinogram Optic Nerve Head Components and Visual Field Defects in Patients with Glaucoma

**DOI:** 10.1155/2015/397495

**Published:** 2015-11-30

**Authors:** Chan Hee Moon, Jungwoo Han, Young-Hoon Ohn, Tae Kwann Park

**Affiliations:** ^1^Department of Ophthalmology, Seoul St. Mary's Hospital, The Catholic University of Korea College of Medicine, Seoul 06591, Republic of Korea; ^2^Department of Ophthalmology, Soonchunhyang University College of Medicine, Bucheon Hospital, Bucheon 14584, Republic of Korea

## Abstract

*Purpose*. To investigate the local relationship between quantified global-flash multifocal electroretinogram (mfERG) optic nerve head component (ONHC) and visual field defects in patients with glaucoma.* Methods*. Thirty-nine patients with glaucoma and 30 normal controls were enrolled. The ONHC amplitude was measured from the baseline to the peak of the second positive deflection of the induced component. The ONHC amplitude was normalized by dividing ONHC amplitude by the average of seven largest ONHC amplitudes. The ONHC amplitude ratio map and ONHC deficiency map were constructed. The local relationship between the ONHC measurements and visual field defects was evaluated by calculating the overlap between the ONHC deficiency maps and visual field defect plots.* Results.* The mean ONHC amplitude measurements of patients with glaucoma (6.01 ± 1.91 nV/deg^2^) were significantly lower than those of the normal controls (10.29 ± 0.94 nV/deg^2^) (*P* < 0.001). The average overlap between the ONHC deficiency map and visual field defect plot was 71.4%. The highest overlap (75.0%) was between the ONHC ratios less than 0.5 and the total deviations less than 5%.* Conclusions.* The ONHC amplitude was reduced in patients with glaucoma compared to that in normal controls. Loss of the ONHC amplitude from the global-flash mfERG showed a high local agreement with visual field defects in patients with glaucoma.

## 1. Introduction

The multifocal electroretinogram (mfERG) technique was developed to provide a topographic measure of retinal activity. It provides objective, noninvasive measures of retinal function loss [1, 2]. The mfERG has been demonstrated to be a valuable test to detect and monitor outer retinal disorders, as well as some inner retinal diseases, such as diabetic retinopathy [3]. However, the responses in conventional mfERGs originate largely from the outer retina and capture relatively little information from the ganglion cells [4]. When the mfERG technique was applied to the study of glaucoma, conventional stimulation was not sensitive enough to detect early stages of glaucoma, which mainly affects retinal ganglion cells and their axons. Although the photopic negative response from the multifocal ERG and second-order kernel responses have been suggested for sensitive detection of inner retinal dysfunction, there is no definite correlation between those responses and visual field defects in glaucoma [5, 6].

Global-flash is a recently introduced modification of the mfERG stimulation paradigms [7–10]. In global-flash protocols, at least one global-flash frame is inserted in the* m*-sequence. Direct component (DC) and induced component (IC) waveforms are then obtained. The DC is the response to the focal flash and is analogous to the conventional mfERG response. The IC is the response to the global flash and reflects the recovery response from the preceding focal flash and is believed to be generated by the inner retina [11]. The IC component is enlarged in the global-flash protocol and the underlying ONHC is responsible for the nasotemporal asymmetry. The ONHC is a component of the electroretinogram that originates from optic nerve fibers and retinal ganglion cell axons at the optic nerve head. It is identified by its implicit time course, which increases with distance along the stimulated area from the optic disc; this is defined as nasotemporal asymmetry. This increase is due to the relatively slow propagation of action potentials along unmyelinated retinal ganglion cell axons [12, 13].

Recently, several glaucoma studies reported their results with the global-flash paradigms and ONHC. Luo et al. noted that losses of nasotemporal asymmetry and low-frequency component power occurred in the global-flash mfERG signals from eyes of nonhuman primates with experimental glaucoma [7, 8]. Fortune et al. demonstrated selective loss of an oscillatory feature in the IC response in patients with glaucoma and suggested that this effect may be due in part to a loss of the ONHC [9]. However, there are no previous studies that quantified the ONHC deficiency in the global-flash response or examined local correlation with visual field defects in human eyes.

In the present study, we quantified the ONHC by measuring the amplitude of the second positive deflection of the IC response waveform, which demonstrates nasotemporal asymmetry. Then, the local relationship between the quantified ONHC and visual field defects obtained by standard automated perimetry (SAP) was investigated.

## 2. Materials and Methods

### 2.1. Subjects

The present study enrolled 39 patients with primary open angle glaucoma (POAG) and 30 age-matched healthy subjects. This study was approved by the Institutional Review Board of Soonchunhyang University Hospital, Bucheon, and conformed to the tenets of the Declaration of Helsinki. Informed consent was obtained from all patients and normal control subjects.

The diagnosis of POAG was based on the presence of a glaucomatous optic disc associated with visual field defects measured by SAP and an open angle confirmed by gonioscopy. The glaucomatous optic disc was determined by a cup/disc ratio of at least 0.5 with typical glaucomatous changes including localized neuroretinal rim thinning. According to the diagnostic criterion for minimal abnormality in the visual field, a visual field defect was determined to be glaucomatous when it met one of three criteria [14]: (1) the pattern deviation plot showed a cluster of three or more nonedge points that had lower sensitivities than 5% of that of the normal population (*P* < 0.05) and one of the points had a sensitivity level that was less than 1% of the population (*P* < 0.01); (2) the value of the corrected pattern standard deviation was less than that of 5% of the normal visual field (*P* < 0.05); or (3) the Glaucoma Hemifield Test showed that the field was outside the normal limits. All patients had a history of a highest measured central corneal thickness adjusted intraocular pressure (IOP) above 21 mm Hg and controlled IOP at the time of testing. The adjusted IOP was calculated by using the following compensation formula; adjusted IOP = measured  IOP + (550 − CCT) × 0.040 [15].

The age-matched controls had no history of chronic ocular or systemic disease and no other pathologic features in a complete ophthalmic examination.

### 2.2. mfERG Recordings

**Figure 1:**
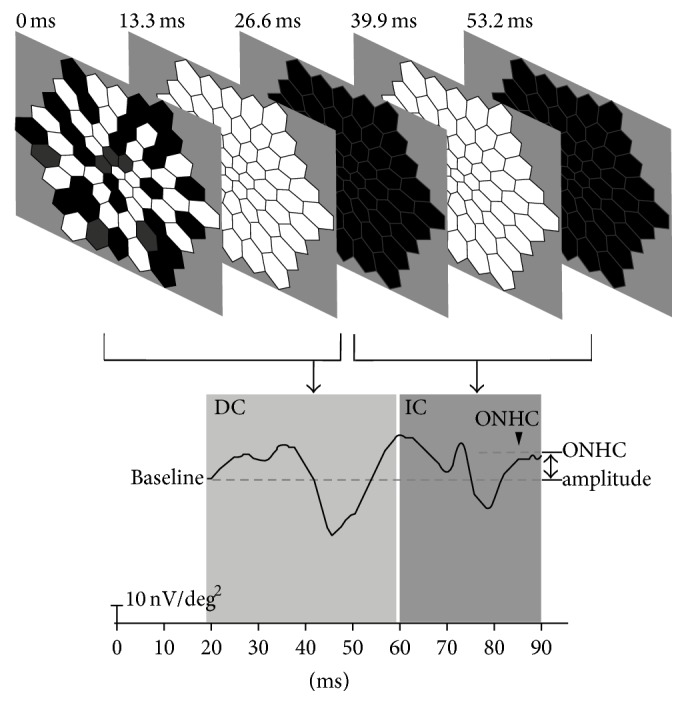
Stimulus sequence of the global-flash multifocal electroretinogram and the resulting response. The global-flash protocol contained a standard* m*-sequence step, followed by a global flash, then a dark frame, another global flash, and lastly a dark frame. The interval for each frame was 13.33 ms. The amplitude from the baseline to the peak of the second positive deflection in IC response epoch which selected from 60–90 ms was recorded as the ONHC amplitude.

The mfERGs were recorded with the VERIS Science 6.4.1 (Visual Evoked Response Imaging System; Electro-Diagnostic Imaging Inc., Redwood City, CA). Pupils were fully dilated with a topical application of 1% tropicamide and 2.5% phenylephrine hydrochloride. Burian-Allen bipolar contact lens electrodes (Hansen Ophthalmic Development Labs, Iowa City, IA) were used, with the ground electrode placed on the earlobe. The stimulus matrix consisted of 61 hexagonal elements. The size of the hexagons was scaled with eccentricity to elicit approximately equal amplitude responses at all locations. At a viewing distance of 27 cm, the radius of the stimulus array subtended approximately 30°. Fixation stability was monitored with a built-in infrared camera. The global-flash protocol contained a standard* m*-sequence step, followed by a global flash, then a dark frame, another global flash, and lastly a dark frame (Figure 1) [7, 8]. The interval for each frame was 13.33 ms. The recording was taken in a room with luminance of 100 cd/m^2^. The maximum luminance of the* m*-sequence and global-flash frames was 200 cd/m^2^, and the dark frames of both stimuli were 15 cd/m^2^. The background luminance of each frame was 55 cd/m^2^. The duration of the data acquisition was about 8 minutes, divided into 16 segments of 30 seconds each. Signals were amplified with a gain of 50,000 and band pass filtered from 10–300 Hz (A Grass PS22, Grass-Telefactor; Astro-Med Inc., West Warwick, RI).

### 2.3. Quantification of ONHC

**Figure 2:**
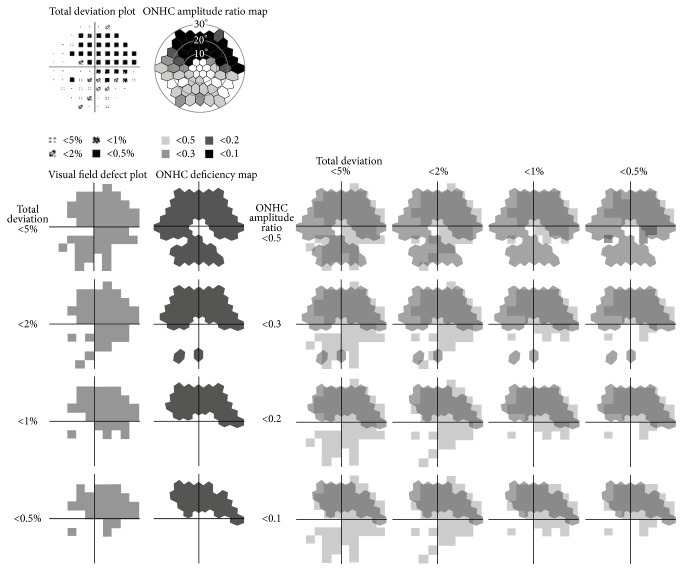
Calculations of overlapping rates between ONHC deficiency maps and visual field defect plots at different stages of functional loss. Areas of visual field defects were extracted from the total deviation plots in each of the four stages: visual sensitivities lower than 5%; lower than 2%; lower than 1%; and lower than 0.5%. ONHC deficiency maps were extracted from the ONHC ratio maps in each of the four stages: ONHC ratio lower than 0.5; lower than 0.3; lower than 0.2; and lower than 0.1. Four visual field defect plots and four ONHC deficiency maps were overlapped for each comparison and the spatial agreement between the ONHC deficiencies and visual field defects was evaluated by calculating rate of overlapping area.

The response epoch comprising the IC was selected, 60–90 ms. The amplitude from the baseline to the peak of the second positive deflection was recorded as the ONHC amplitude, at 61 hexagons. The amplitude of the initial DC response at 20 ms was set as the baseline (Figure 1). The ONHC ratio was calculated to normalize the responses by dividing the measured ONHC amplitudes by the average of the seven largest ONHC amplitude measurements among the 61 hexagons. Seven hexagonal elements were selected from within ring 3 and most were chosen from rings 1 and 2. ONHC deficiency was defined as an ONHC ratio less than 0.5. ONHC deficiency was divided into four stages: between 0.5 and 0.3; between 0.3 and 0.2; between 0.2 and 0.1; and less than 0.1. ONHC ratio maps were constructed using gray scale according to the calculated values. Then, ONHC deficiency maps were extracted from the ONHC ratio maps (Figure 2).

### 2.4. Automated Perimetry

Automated perimetry was conducted using the Central 30-2 Swedish Interactive Threshold Algorithm (SITA) with a Humphrey visual field Analyzer II (Carl Zeiss Meditec, Dublin, CA) and a Goldmann size III stimulus on a 31.5-apostilb background. Areas of visual field defects were extracted from the total deviation (TD) plots in each of the four stages: visual sensitivities lower than those of 5% of the normal population; lower than 2%; lower than 1%; and lower than 0.5% (Figure 2).

### 2.5. Evaluation of Local Relationship between ONHC Deficiency and Visual Field Defect

61 hexagonal arrays of mfERGs and total deviation plots of SAP, both covering central 30 degrees of the visual field each, were matched in the same image size. Next, four visual field defect plots and four ONHC deficiency maps were overlapped for each comparison (Figure 2). The local relationship between the ONHC deficiencies and visual field defects was evaluated by the overlap. The overlap between an ONHC deficiency map and visual field defect plot was calculated as follows: [(intersection area between the ONHC deficiency map and visual field defect plot) × 2]/(sum of the area of ONHC deficiency and visual field defect plot), (% presentation). Image processing and area measuring were conducted using imaging software (ImageJ 1.43u, Wayne Rasband, National Institutes of Health, available at http://rsb.info.nih.gov/ij/index.html).

### 2.6. Statistical Analysis

An independent *t*-test was performed to compare both the averages of the seven largest ONHC amplitudes between patients with glaucoma and normal controls. Differences in overlap between the visual field defects and ONHC deficiencies across various stages were evaluated by one-way analysis of variance (ANOVA) using a post hoc Bonferroni test. Statistical analysis was conducted using SPSS Statistics Version 21 (IBM Corporation, Somers, NY). All tests were two tailed and *P* < 0.05 was considered statistically significant.

## 3. Results

### 3.1. General Characteristics

The average age of the patients with glaucoma was 50.83 ± 13.70 years, and it was 49.25 ± 12.80 years for the normal controls. The mean deviation (MD) was −8.44 ± 5.07 in patients with glaucoma and −0.08 ± 0.39 for normal controls (*P* < 0.001). Pattern standard deviation (PSD) was 8.72 ± 4.69 in patients with glaucoma and 1.43 ± 0.27 in normal controls (*P* < 0.001) (Table 1).

### 3.2. ONHC Measurement in Normal and Glaucoma Groups

The mean ONHC amplitude from the 61 hexagons was 6.01 ± 1.91 nV/deg^2^ in patients with glaucoma and 10.29 ± 0.94 nV/deg^2^ in normal controls. The difference was significant (*P* < 0.001). However, the average of the seven largest ONHC amplitudes was 14.6 ± 4.01 nV/deg^2^ in patients with glaucoma and 16.91 ± 2.99 nV/deg^2^ in normal controls. The difference was not significant (*P* = 0.104) (Table 1). Cut-off values of ONHC amplitude of *p* < 5%, *p* < 2%, *p* < 1%, and *p* < 0.5% were 1.73, 0.94, 0.62, and 0.44 nV/deg^2^, respectively. Cut-off values of ONHC ratio of *p* < 5%, *p* < 2%, *p* < 1%, and *p* < 0.5% were 0.10, 0.63, 0.62, and 0.55, respectively, on 30 healthy volunteers. ONHC amplitude measurements and calculated ONHC ratio were demonstrated in Table 2.

### 3.3. Local Relationship between ONHC Deficiencies and Visual Field Defects

**Figure 3:**
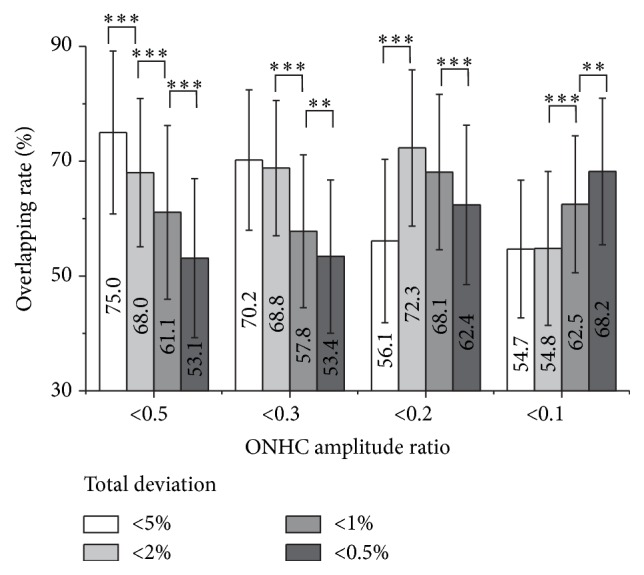
Overlapping rates between total deviation and optic nerve head component ratios. ^*∗∗*^ *P* < 0.01, ^*∗∗∗*^ *P* < 0.001.

The ONHC ratios less than 0.5 showed the highest local agreement with the total deviation of visual sensitivities less than 5%. The overlap was 75.0 ± 14.21%. The ONHC ratios <0.3 highly correlated with both TD <5% and <2%. The overlaps were 70.2 ± 12.22% for the less than 5% group and 68.8 ± 11.77% for the less than 2% group. The difference of overlap between the TD <5% group and the <2% group was not significant (*P* = 0.139). The ONHC ratio <0.2 showed high agreement with both TD <2% and <1%. Overlaps were 72.3 ± 13.60% and 68.1 ± 13.54%. The difference of agreement rates between TD <2% and <1% was not significant (*P* = 0.094). The ONHC ratio <0.1 showed the best agreement with TD <0.5%. The overlap was 68.2 ± 12.77% (Table 3, Figure 3).

## 4. Discussion

**Figure 4:**
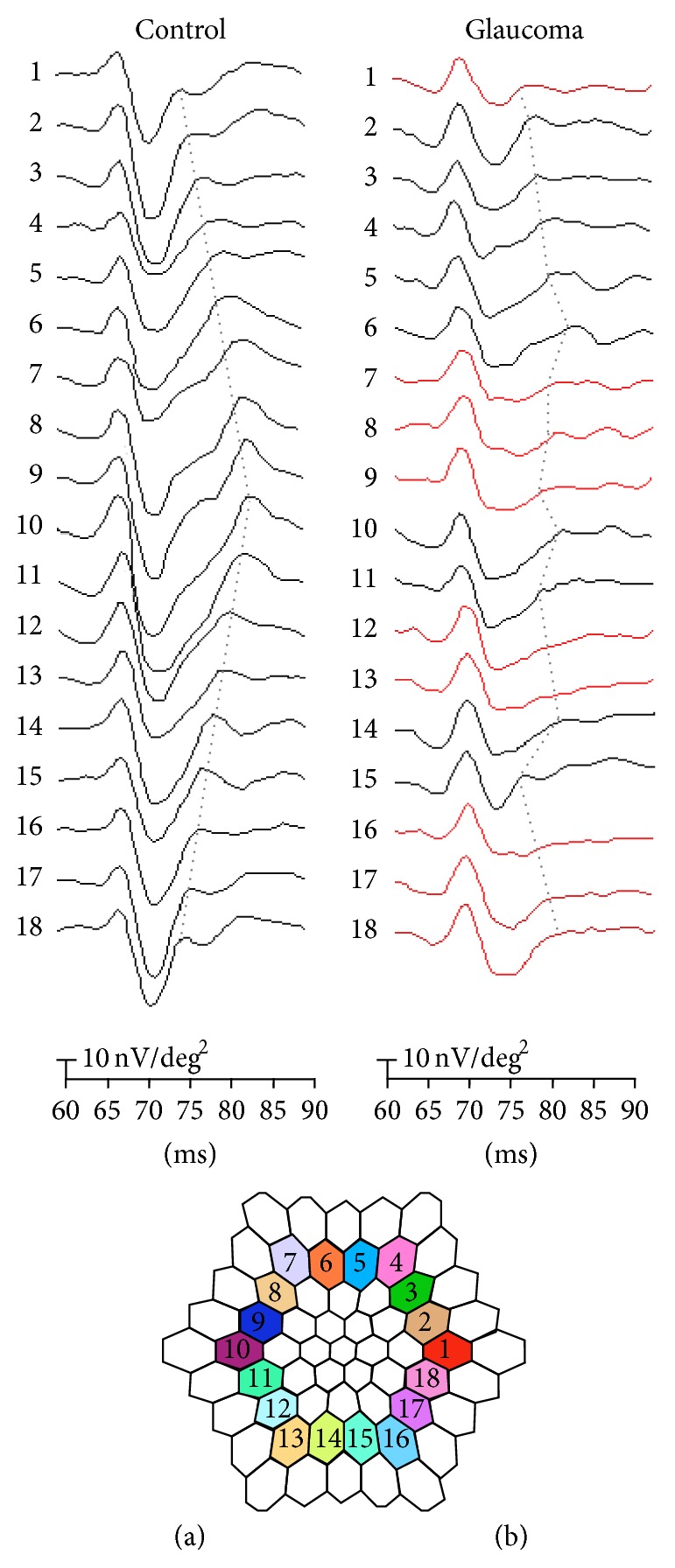
Induced component waveforms in normal controls and patients with glaucoma. IC of normal controls (a) shows intact second positive deflection waveform and nasotemporal asymmetry. IC of patients with glaucoma (b) shows reduced second positive deflection in some waveforms (red line) and disrupted nasotemporal asymmetry. Dot line indicates optic nerve head components.

In the present study, we measured ONHC amplitudes obtained from global-flash mfERGs and investigated the local relationship between the ONHC amplitudes and visual field defects in patients with glaucoma. The ONHC is identified by its implicit time course, which increases with distance along the stimulated area from the optic disc which is called nasotemporal asymmetry [12, 13]. It has been reported that pharmacological suppression of inner retinal activity by intravitreal injection of tetrodotoxin (TTX, a blocker of sodium-channel dependent action potentials) reduced or eliminated the nasotemporal asymmetry in fast flicker mfERGs [16–18]. The nasotemporal asymmetry is augmented by the global-flash protocol [8, 19]. There are different ways of inserting the global flash in the* m*-sequence, and the aim is to enhance the adaptive effect of the retina to obtain the inner retinal contribution [20]. The IC of the global-flash mfERG response waveform resembles higher order kernels of the fast flicker mfERG (in terms of interflash interaction) and is believed to be generated by the inner retina [11, 21]. The ONHC can be refined by extracting the signal with the Sutter and Bears time-domain algorithm [12]; however, application of this multistep mathematical algorithm to extract the component is somewhat complicated in practical use. In this study, we quantified the ONHC by directly measuring the amplitude of the second positive deflection of the IC response waveform that comprised the nasotemporal asymmetry. The amplitude of the second positive deflection of the IC is representative of ONHC activity. The reduction of the second positive deflection of the IC response waveform with the global-flash paradigm implies a loss of the nasotemporal asymmetry (Figure 4); this loss of nasotemporal asymmetry reflects the loss of the ONHC. Therefore, the reduction of the second positive deflection of the IC response can demonstrate a loss of ONHC.

The mean ONHC amplitude measurements from the patients with glaucoma were significantly lower than that of normal controls. This result is in keeping with previous studies of experimental glaucoma, which showed a decrease of nasotemporal asymmetry after intravitreal TTX injections [16–18]. However, the average of the seven largest ONHC amplitudes between patients with glaucoma and normal controls was not significantly different, though normal controls have slightly higher values. This result may be due to the moderate disease severity of the enrolled patients and the characteristics of glaucomatous visual field loss. The average MD of glaucoma patients was −8.44. All of the glaucoma patients had preserved central 5 to 10 degrees of visual field sensitivity. Central 10 degrees of the visual field correspond to rings 1-2 of mfERG, and rings 1-2 are composed of seven hexagonal elements. Therefore, we use the average value of seven hexagonal elements as reference for normalization in each subject. Relative central visual field conservation and preserved central mfERG responses were responsible for the similar large ONHC amplitudes values between patients with glaucoma and normal controls.

**Figure 5:**
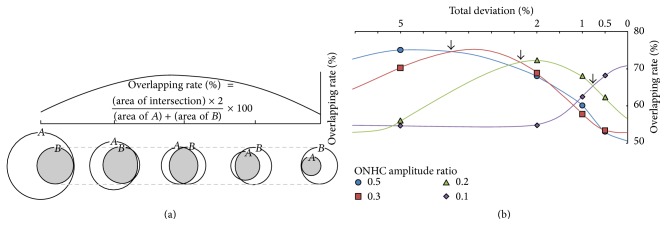
(a) Quadric curve of overlap. (b) Estimated overlap curve between total deviation and optic nerve head component ratios. When one area is fixed and the other area changes, the overlapping rate values necessarily demonstrate a negative quadric curve (a). The highest correspondence was between ONHC ratios <0.5 and TD <5%, ONHC ratio <0.3 and TD <3 or 4%, ONHC ratio <0.2 and TD <2 and 1%, and ONHC ratio <0.1 and TD <0.5% (b).

Loss of the ONHC amplitude showed a high local agreement with visual field defects. The average overlap between the ONHC deficiency map and the visual field defect plot was 71.42% across the different stages of functional loss. The highest agreement was between the ONHC ratio <0.5 and TD <5%; the ONHC ratio <0.2 and TD <2% and 1%; and the ONHC ratio <0.1 and TD <0.5%. The ONHC ratio <0.3 showed high agreement with TD <5% and 2%. However, the overlaps were smaller than that of the ONHC ratio <0.5 and TD <5% (70.2% versus 75.0%) and the ONHC ratio <0.2 in TD <2% (68.8% versus 72.3%). When one has fixed area and the other has changed, the overlap necessarily demonstrates a negative quadric curve (Figure 5). When one area is fixed and the other area becomes larger, the intersection area increases but the overlap decreases. In contrast, when one area is fixed and the other area becomes smaller, the intersection area decreases and the overlap subsequently decreases. The vertex value is given when the two other areas completely overlap more in size. Therefore, when estimating the quadric curve of the overlap between the ONHC ratios less than 0.3 and visual field defects, the vertex value is presumed to be a total deviation of 3 or 4% (Figure 5). Namely, the ONHC ratio less than 0.3 may show the best agreement with a total deviation less than 3 or 4%.

There were a number of limitations to this study. The 61-hexagon stimulus array was scaled to obtain equal amplitude responses. The array used for the perimetry was not scaled, and hence hexagons at different locations contained different numbers of visual field test points. For this reason, we compared the total area of the visual field defects and ONHC deficiencies to reduce the inaccuracy of the one to one correspondence, rather than compare the hexagon elements and visual field test points by matched pairs.

Patients with visual field defects are more likely to have electrophysiological abnormalities than patients with preperimetric glaucoma. Further investigations are required for evaluating the usefulness of ONHC amplitude from the global-flash mfERG in patients with preperimetric glaucoma.

The ONHC amplitude measurements varied considerably from person to person; therefore, a normal value was not established. In the present study, ONHC ratios were used for analysis to reduce the interpersonal variation. This study enrolled the patients with mild to moderate glaucoma and subjects had preserved central visual field sensitivity and good mfERGs response. The averages of the seven largest ONHC amplitudes between glaucoma patients and normal controls were not significantly different. However, if the mfERG responses from every hexagonal element were proportionally reduced in advanced glaucoma, the normalization using an intraindividual ratio format would not provide any useful information. In such a case, normalization using a normal range value obtained from controls of large population will be needed.

The main way for evaluating functional loss of visual field in glaucoma is a perimetry. Perimetry is based on psychophysics and is a subjective measurement. Although perimetry is an excellent tool, there is always a need for determining the functional loss with objective method. Electroretinogram, involving mfERG, is one of the objective tools. However, conventional mfERG mostly reflects outer retinal function and is not suitable for early detection of glaucoma. In this study, we used global-flash mfERG protocols, which are more targeted at the inner retinal function. Even more, we directly measured the second positive deflection amplitude of the IC as ONHC for easier practical use and evidenced a significant agreement between ONHC amplitude loss and visual field defect in SAP. We imply that ONHC amplitude can be used as objective tool for evaluating functional loss in glaucoma, with further investigations.

## 5. Conclusions

The ONHC amplitude was reduced in patients with glaucoma compared to normal controls. Loss of global-flash mfERG ONHC amplitudes showed a high local agreement with visual field defects obtained by SAP in patients with glaucoma. The ONHC amplitude measurements from global-flash mfERG serve as an objective method to evaluate local functional loss in patients with glaucoma. The overlap was greatest when the largest range of severity for the deviation and amplitude reductions were compared.

## Figures and Tables

**Table 1:** Demographic characteristics.

Variable	Normal	Glaucoma	*P* value
Number	30	39	
Age	49.25 ± 12.80	50.83 ± 13.70	0.858
Sex ratio (M/F)	0.60	0.85	0.235
MD (dB)	−0.08 ± 0.39	−8.44 ± 5.07	<0.001
PSD (dB)	1.43 ± 0.27	8.72 ± 4.69	<0.001
Average ONHC amplitude (nV/deg^2^)	10.29 ± 0.94	6.01 ± 1.91	<0.001
Average of seven largest ONHC amplitudes (nV/deg^2^)	16.91 ± 2.99	14.60 ± 4.01	0.104

**Table 2:** ONHC amplitude measurements and calculated ONHC ratio.

	Normal		Glaucoma	
	ONHC amplitude (nV/deg^2^)	ONHC ratio	ONHC amplitude (nV/deg^2^)	ONHC ratio
Ring 1	16.90 ± 2.99	0.95 ± 0.25	14.33 ± 4.25	0.98 ± 0.21
Ring 2	12.27 ± 5.07	0.67 ± 0.32	7.57 ± 5.12	0.52 ± 0.31
Ring 3	10.69 ± 3.72	0.60 ± 0.29	6.49 ± 5.24	0.43 ± 0.32
Ring 4	8.04 ± 3.26	0.43 ± 0.38	3.98 ± 3.32	0.28 ± 0.22

**Table 3:** Overlap between total deviation and optic nerve head component ratios.

Total deviation	Optic nerve head component ratio			
	<0.5	<0.3	<0.2	<0.1
<5%	75.0 ± 14.21	70.2 ± 12.22	56.1 ± 14.23	54.7 ± 12.00
*P* value	<0.001	0.139	<0.001	0.877
<2%	68.0 ± 12.92	68.8 ± 11.77	72.3 ± 13.60	54.8 ± 13.41
*P* value	<0.001	<0.001	0.094	<0.001
<1%	60.1 ± 15.12	57.8 ± 13.31	68.1 ± 13.54	62.5 ± 11.93
*P* value	<0.001	0.005	<0.001	0.001
<0.5%	53.1 ± 13.85	53.4 ± 13.35	62.4 ± 13.87	68.2 ± 12.77
