# Corrigendum to “Local Relationship between Global-Flash Multifocal Electroretinogram Optic Nerve Head Components and Visual Field Defects in Patients with Glaucoma”

**DOI:** 10.1155/2016/8592790

**Published:** 2016-06-28

**Authors:** Chan Hee Moon, Jungwoo Han, Young-Hoon Ohn, Tae Kwann Park

**Affiliations:** Department of Ophthalmology, Soonchunhyang University College of Medicine, Bucheon Hospital, Bucheon 14584, Republic of Korea

In the article titled “Local Relationship between Global-Flash Multifocal Electroretinogram Optic Nerve Head Components and Visual Field Defects in Patients with Glaucoma” [1] the affiliation of the first author was incorrectly listed as “Department of Ophthalmology, Seoul St. Mary's Hospital, The Catholic University of Korea College of Medicine, Seoul 06591, Republic of Korea.” The correct affiliations are presented above.
